# Nanostructure and Morphology of the Surface as Well as Micromechanical and Sclerometric Properties of Al_2_O_3_ Layers Subjected to Thermo-Chemical Treatment

**DOI:** 10.3390/ma15031051

**Published:** 2022-01-29

**Authors:** Marek Bara, Mateusz Niedźwiedź, Władysław Skoneczny, Adrian Barylski

**Affiliations:** Faculty of Science and Technology, Institute of Materials Engineering, University of Silesia in Katowice, 41-500 Chorzów, Poland; mateusz.niedzwiedz@us.edu.pl (M.N.); wladyslaw.skoneczny@us.edu.pl (W.S.); adrian.barylski@us.edu.pl (A.B.)

**Keywords:** aluminium, anodic oxide, thermo-chemical treatment, nanostructured oxide, microhardness, adhesion

## Abstract

The article presents the effect of the thermo-chemical treatment of Al_2_O_3_ layers on their nanostructure, surface morphology, chemical composition as well as their micromechanical and sclerometric properties. Oxide layers were produced on EN AW-5251 aluminium alloy (AlMg_2_) by the method of direct current anodizing in a three-component electrolyte. The thermo-chemical treatment was carried out in distilled water and aqueous solutions of Na_2_SO_4_·10H_2_O and Na_2_Cr_2_O_7_·2H_2_O. It was shown that the thermo-chemical treatment process changes the morphology of the surface of the layers (the formation of a sub-layer from the Na_2_SO_4_·10H_2_O and Na_2_Cr_2_O_7_·2H_2_O solutions), which directly increases the thickness of the layers by 0.37 and 1.77 µm, respectively. The thermo-chemical treatment in water also resulted in the formation of a 0.63 µm thick sub-layer. The micromechanical tests indicated a rise in the surface microhardness of the layers in the case of their thermo-chemical treatment in water and the Na_2_SO_4_·10H_2_O solution and a decrease in the case of the layers modified in the Na_2_Cr_2_O_7_·2H_2_O solution. The highest microhardness (7.1 GPa) was exhibited by the layer modified in the Na_2_SO_4_·10H_2_O solution. Scratch tests demonstrated that the thermo-chemically treated layers had better adhesive properties than the reference layer. The best scratch resistance was exhibited by the layer after thermo-chemical treatment in the Na_2_SO_4_·10H_2_O solution (the highest values, practically for all the critical loads) which, together with its low roughness and high load capacity, predispose it to sliding contacts.

## 1. Introduction

Among other things, in order to increase the hardness and abrasion resistance of an aluminum surface layer, oxide layers are formed on its surface. The most effective method of aluminium surface protection is the electrochemical method of anodic oxidation and hard anodizing [1,2]. Aluminium oxide layers produced by these methods are characterized by a specific nano-structure [3,4]. The electrochemical anodizing process of aluminium and its alloys, despite the fact that it was developed in the 1920s, is still one of the optimal and most frequently employed processes for the surface protection of aluminium elements. The choice of the method of aluminium surface protection depends mainly on its intended use. Al_2_O_3_ layers intended for sliding contacts are produced by hard anodizing [5]. Typical hard anodizing is performed using electrolytes with an average ability to re-dissolve, the most popular of which are aqueous solutions of C_2_H_2_O_4_ or H₂SO₄ [6,7]. The disadvantage of these electrolytes is the need for intensive cooling of the bath owing to the rapid rise in temperature in the anode area resulting from the electrical resistance of the layer being formed. The layers produced in aqueous solutions of C_2_H_2_O_4_, H₂SO₄ acids at elevated temperatures exhibit high porosity and low adhesion to the substrate due to the process of secondary dissolution of the layer. The solution to this problem is to use an electrolyte with active organic substances that inhibit the process of secondary dissolution of the layer. The functions of such substances can be performed by organic dicarboxylic acids [8]. The method of hard anodizing of aluminium alloys carried out in three-component electrolytes allows Al_2_O_3_ layers for tribological applications to be obtained, even at temperatures above 303 K. The sclerometric studies of the Al_2_O_3_ layers showed a linear dependence of the increase in the thickness of the oxide layers with the increase in the diameter of the nanofibers. Al_2_O_3_ layers of small thickness and diameter of nanofibers are subject to grooving. The increase in production parameters, and hence the thickness of the layers, changes the wear process of the layers from grooving to drawing and micro-cutting [3]. Oxide layers produced by the above methods are applied primarily in lubricated and oil-free sliding contacts of working machines such as compressors, actuators, shock absorbers and motors.

The thermo-chemical treatment of oxide layers is based on the effect of heat and a chemically active medium on their surface. The purpose of this interaction is to change the nanostructure and chemical composition of the layers by saturating them with an appropriate chemical compound [9]. The most commonly employed substances used in the thermo-chemical treatment of oxide layers are distilled water and steam. The effectiveness of the thermo-chemical treatment is influenced by the time of operation, temperature and pH of the bath. The oxide layers are hygroscopic and able to absorb a significant amount of water. As the temperature increases, this process increasingly becomes irreversible, and above 343 K the reaction becomes irreversible. The thermo-chemical treatment process is most appropriate in baths, the pH of which is within the range of 5.5–6.5 with a low content of calcium and magnesium salts. At lower pH values, the layer is completely sealed and partially etched. In more alkaline baths, the surface of the layer becomes mechanically less durable. Therefore, it is recommended to use distilled water [10]. The process of thermo-chemical treatment of oxides can take place at different rates. It occurs faster the higher the temperature is. Usually the process is carried out at a temperature of not less than 368 K. At temperatures close to 473 K, the process is very fast in the first 2 min. The process time also depends on the composition of the electrolyte, the thickness of the oxide layer and the anodizing conditions [11]. The use of steam is the most ecological method; however, it reduces the hardness of the layers as a result of their hydration (increasing the γ-AlOOH phase) [12,13]. Researchers have also applied other compounds for the thermo-chemical treatment of oxide layers. Lanthanide salts were used in works [14,15,16]. In the multi-step Ce-Mo method, the treatment is performed in Ce(NO_3_)_3_ and CeCl_3_ solutions with re-anodizing in an Na_2_MoO_4_ solution between the processes. In another method using NaCl-SnCl_2_-CeCl_3_ salt, the treatment is conducted at the temperature of 473 K for 2 h. These methods give particularly good results in anti-corrosion protection, but are quite time-consuming and energy-consuming. Thermo-chemical treatment carried out in nickel fluoride solutions [17,18,19] also gives good results in anti-corrosion protection. It is performed in 5–15 min, at temperatures of 303–323 K. Its advantage is the reduction of energy costs compared to the methods conduced with lanthanide salts; nevertheless, similar to the treatment with steam, a significant reduction in the hardness of the layers is obtained, associated with the appearance of the γ-Al(OH)_3_ phase. The thermo-chemical treatment of oxide layers employing the above methods is a procedure that primarily protects the base of the layers against corrosion. The process consists in closing the porous oxide layer, which protects the base metal against the effects of many aggressive environments [20]. 

Thermo-chemical treatment carried out in order to increase the wear resistance of the oxide layers should be performed in conditions where the nanostructure of the layers will not be hydrated too much and in chemical compounds resulting in the formation of a sub-layer with good sliding properties. There is no information in the literature on the impact of Al_2_O_3_ layer modification as a result of thermo-chemical treatment with an Na_2_Cr_2_O_7_ or Na_2_SO_4_ solution on the structure, surface morphology, as well as the micromechanical and sclerometric properties. The research presented below can, therefore, be considered innovative.

## 2. Materials and Methods

### 2.1. Research Material

The base material in the anodizing process was the aluminium alloy EN AW-5251. This alloy was chosen owing to its good mechanical properties and the low content of admixtures of other elements, which facilitates the growth of Al_2_O_3_ layers. The layers were produced on plates with an area of 5 × 10^−4^ m^2^ cut with a stream of water from rolled steel 1 × 10^−3^ m thick. The surfaces of the anodized plates were etched in a 5% KOH solution for 20 min and neutralized in a 10% HNO_3_ solution for 5 min. The processes were carried out at room temperature. The plates were rinsed in distilled water after each process. The oxide layers were created in the DC anodizing process using a GPR-25H30D power supply. The anodizing process was conducted in an electrolyte consisting of an aqueous solution of C_2_H_2_O_4_·2H_2_O (30 g/L), C_8_H_6_O_4_ (76 g/L) and an 18% H_2_SO_4_ solution (33 mL/L). During anodizing, the electrolyte was stirred by means of a mechanical stirrer. The cathode in the anodizing process was a lead plate. The parameters of the input variables (current density *j* = 3 A/dm^2^, electrolyte temperature *T* = 298 K, process time *t* = 60 min) were constant for all the samples, and their values were selected as the most appropriate for the production of layers intended for sliding contacts on the basis of preliminary tests. The anodizing process was completed with a 60-min rinse in distilled water. The produced layers were subjected to thermo-chemical treatment in distilled water, Na_2_SO_4_·10H_2_O and Na_2_Cr_2_O_7_·2H_2_O solutions. Distilled water with a pH of 6–7 was applied for the thermo-chemical treatment. The composition of the used bath was Na_2_SO_4_·10H_2_O (200 g/L of distilled water), with a pH of 6–7. Na_2_Cr_2_O_7_·2H_2_O (50 g/L of distilled water) was used to prepare the last bath; the pH of the solution was 8–9. The substances for the thermo-chemical treatment were selected as chemical compounds of various densities. The density of the compounds for the thermo-chemical treatment was: 0.998 g/cm^3^ for distilled water, 1.46 g/cm^3^ for Na_2_SO_4_·10H_2_O and 2.52 g/cm^3^ for Na_2_Cr_2_O_7_·2H_2_O. Table 1 shows the values of the input variables during the thermo-chemical treatment process.

The thermo-chemical treatment process was completed with rinsing in distilled water and allowing each sample to dry. 

### 2.2. Research Methodology

The surface morphology and nanostructure studies of all the layers, along with microanalysis of the chemical composition by EDS, were performed using a JEOL JSM 7200F high-resolution scanning electron microscope (SEM), equipped with an EDS detector (Octane Elite Super), (Jeol, Akishima, Japan). In order to properly observe the layers during the SEM examinations, the samples for surface morphology were sputtered with a layer of gold with a thickness of 5 nm. Anodic oxide layers are poorly conductive, so they charge electrically during operation of the electron beam, which contributes to an incorrect observation. The gold was sputtered by means of a Leica ACE 200 automatic sputtering machine (Leica, Wetzlar, Germany) with a sputtering current of 40 mA. At the first stage of preparation, samples for cross-section investigations were sprayed with gold up to a thickness of 150 nm in order to protect the surface. The spraying was performed employing a JVG-N1 device (Jeol, Akishima, Japan). Then the samples were cut and embedded in conductive epoxy resin. Further preparation of the cross-sectional metallographic specimens was done with sandpaper grades 240, 400, 600, and 1000. Polishing was carried out using polishing cloths by adding a diamond suspension of 3 µm, and then 1 µm. The polishing time was 6 min. Final polishing was performed with a Fischione Model 1051 ion polisher. The polishing voltage was 6 KeV and was performed for 6 h. After the polishing process, ion etching was carried out using the same machine and same voltage as for the polishing. The ion etching time was 30 min. The prepared samples were subjected to SEM examinations. Observation of the surface and nanostructure as well as the registration of micrographs at magnifications of 30,000× and 8000× magnification were performed. Additionally, microanalysis of the chemical composition was conducted by EDS. The microscopic observations of the scratches performed during the sclerometric examinations were undertaken with a Hitachi S-4700 scanning electron microscope (Hitachi High-Tech Corporation, Tokyo, Japan). For proper observation, the layers were sprayed with carbon by means of a turbomolecular carbon sputtering machine. Observation of the surface and the registration of micrographs were conducted at 80× and 35× magnification.

The measurements of the thickness of the sub-layer obtained as a result of the thermo-chemical treatment were made on micrographs of the cross-sections with a magnification of 80× in the ImageJ 1.5 graphics program (Wayne Rasband, MD, USA). Ten measurements of the thickness of the sub-layer over the entire width of the micrograph were taken, then the mean values and the standard deviation were calculated. 

The surface roughness parameters of the layers were determined by the 3D method utilising a KEYENCE VR-6000 Series optical profilograph (KEYENCE INTERNATIONAL Mechelen, Belgium).

The micromechanical tests were performed using a Micro Combi Tester-MCT3 (Anton Paar, Corcelles-Cormondrèche, Switzerland). A Berkovich diamond indenter (B-V 83) was employed, with maximum load of 250 mN, loading and unloading time after 30 s, and holding time under maximum load of 10 s. Three impressions were made for each sample. The *H_IT_* hardness and *E_IT_* modulus of elasticity were determined by the Oliver–Pharr method [21]. The measurements were taken in accordance with the ISO 14577 recommendations [22]. Based on the recorded load-unload curves, the values of total indentation work *W_tot_* and its components (plastic deformation work *W_pl_* and elastic deformation work *W_el_*) were also determined. Additionally, the percentage share of the work of elastic recovery *η_IT_* was determined.

The scratch resistance of the layers was investigated with the Micro Combi Tester-MCT^3^ from Anton-Paar (Corcelles-Cormondrèche, Switzerland) applying the scratch test method. The tests were carried out in accordance with the ISO 20502 [23] and ASTM C1624 [24] guidelines using a Rockwell diamond indenter (Anton Paar, Corcelles-Cormondrèche, Switzerland) with a diameter of 100 μm. Each test was divided into three stages. In the first stage (pre-scan), the profile of the sample was scanned under a load of 0.03 N. In the second stage (scan), the appropriate tests were performed with a progressively increasing load from 0.03 to 30 N. The length of each scratch was 3 mm, with the indenter movement speed of 6 mm/min. The last stage (post-scan) was to measure the profile that was created after making the scratch. The following parameters were recorded: pressure force-*F_n_* [N], friction force-*F_t_* [N], *P_d_*-penetration depth of the indenter under load [μm], *R_d_*-penetration depth after unloading [μm], and *AE*-acoustic emission [%]. For all the layers, three critical loads were determined: *Lc1* (the critical load at which the first damage of the studied layers occurred—Hertz tensile cracks inside the scratch mark), *Lc2* (the critical load at which the first cohesive damage of the layers appeared) and *Lc3* (the load, at which the layers were completely damaged).

## 3. Results and Discussion

### 3.1. Surface Morphology of Layers after Thermo-Chemical Treatment

**Figure 1:**
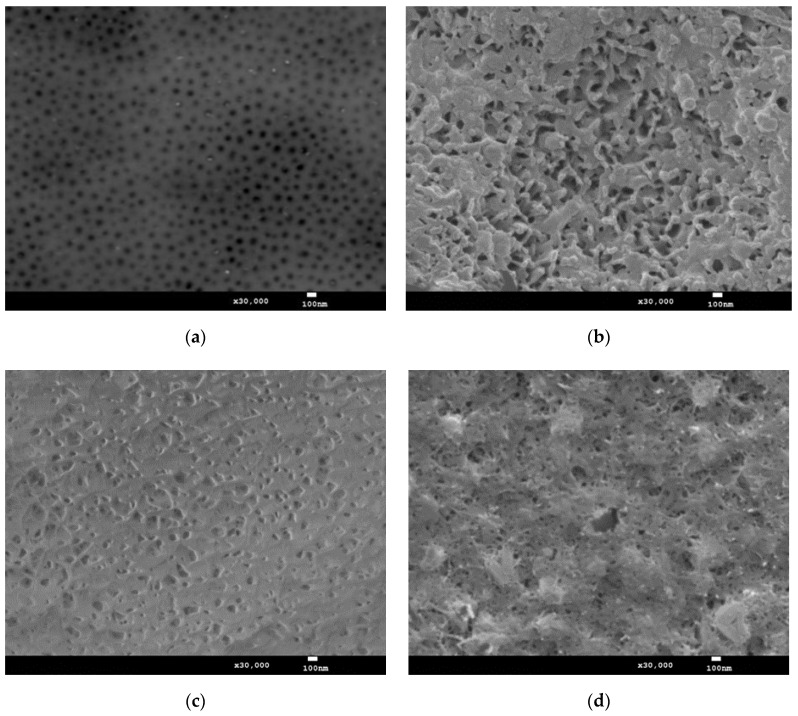
Surface morphology of layers: (**a**) Sample 1, (**b**) Sample 2, (**c**) Sample 3, (**d**) Sample 4.

The microscopic examinations revealed characteristic surface porosity for the reference layer, the elements of which are micro- and nanopores (Figure 1a). Micropores arise as a result of disturbances in the structure caused by the transfer of substrate defects to the surface of the oxide layer [25]. The nanopores, in turn, are formed by the contact of the oxide fibers and are present in all the cross-sections of the fibers throughout the thickness of the layer. The porosity of Al_2_O_3_ layers produced in ternary electrolytes depends mainly on the length of the aliphatic chain in dicarboxylic acids and the temperature of the electrolyte [26]. Its growth in the vicinity of the anode causes an increase in the secondary dissolution of aluminium oxide and, as a result, a layer with increased porosity is obtained.

(Figure 1b–d) show significant or complete sealing of the characteristic aluminium oxide nanopores by the products of the applied treatment, regardless of the solutions applied in the thermo-chemical treatment process. The nature of the surface morphology of the layers after thermo-chemical treatment is related to the formation of a new phase (a sub-layer). The layer after the Na_2_SO_4_·10H_2_O process (Figure 1c) is shown to be more homogeneous and evenly built-up with the thermo-chemical treatment products. The sub-layer has the character of planar multidirectional sticky fibers with unevenly positioned pores. The sub-layers produced in the process with the H_2_O distillate (Figure 1b) and Na_2_Cr_2_O_7_·2H_2_O (Figure 1d) show a slightly different nature of the surface. Their participation consists of precipitates forming a spatial mesh, which is the reason for the higher roughness of these layers, confirmed by the investigation of the geometrical structure of the surface. 

### 3.2. Nanostructure of Layers after Thermo-Chemical Treatment

The nanostructure of the Al_2_O_3_ layers shows a fibrous structure, oriented along the development of the layer due to the influence of the electric field. The aluminium oxide fibers create free spaces between themselves, which are a channel for the migration of oxygen ions connecting with the anode material in the electrochemical process. There are three stages of the growth of the oxide layer. The first is the transition of Al^3+^ ions from the metal to the oxide layer. The second is the diffusion of Al^3+^ ions through the “barrier layer.” The third is the oxidation (film-forming) reaction. As a result of the different rates of formation, the Al_2_O_3_ layer on the metal side has an excess of Al^3+^ ions located in the oxide structure. On the electrolyte side, there is an excess in the layer. oxygen ions. Parallel alignment of the fibers of the layers is obtained only when using a flat, very clean substrate. The use of aluminium alloys as the base of the Al_2_O_3_ layer and etching of its surface cause slight distortion of the oxide nanostructure visible in the micrographs.

**Figure 2:**
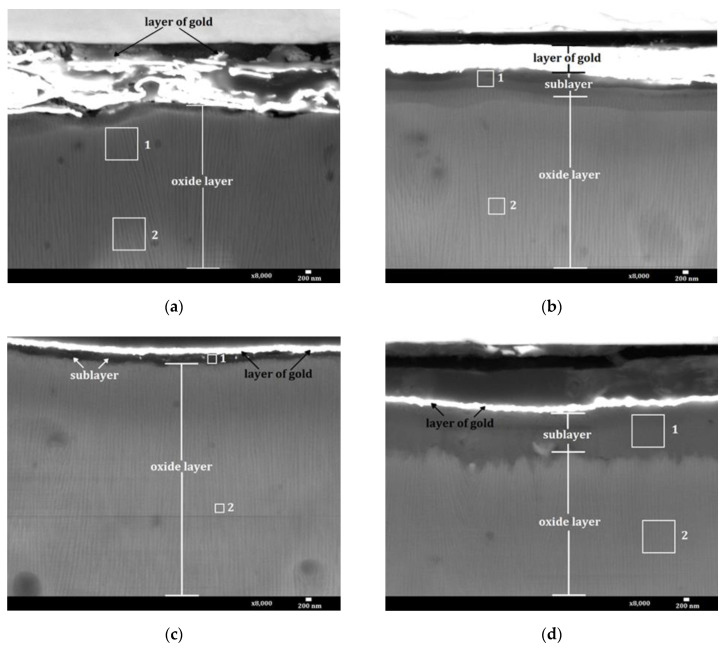
Nanostructure of layers: (**a**) Sample 1, (**b**) Sample 2, (**c**) Sample 3, (**d**) Sample 4.

In all the micrographs, the layer of gold that was sputtered at the first stage of preparation in order to protect the surface, is visible at the top. The gold was sputtered to a thickness of 150 nm; however, only in two micrographs (Figure 2c,d) is the layer of this thickness. On the other two cross-sections (Figure 2a,b), delamination and smearing of the sprayed material occurred, and as a result these gold layers seem much thicker. In the case of the thermo-chemically treated oxide layers (Figure 2b–d), the sub-layer produced by the process is visible between the gold layer and the oxide surface. Depending on the compound used in the thermo-chemical process, different thicknesses of the sub-layer were obtained (Table 2).

The thickest sub-layer (about 1.77 µm) was obtained using Na_2_Cr_2_O_7_·2H_2_O for the thermo-chemical treatment. The thickness of the sublayer made with distilled water was about 0.63 µm, while the sublayer made using Na_2_SO_4_·10H_2_O had the smallest thickness of about 0.37 µm. The differences in the thickness of each sub-layer resulted only from the employed chemical compound because the individual processes were carried out with the same process parameters. 

### 3.3. Elemental Composition of Layers

The results of the chemical composition tests are presented in Table 3. The percentage content of a given element was determined from the areas marked as 1 and 2 on the cross-sections of the layers (Figure 2). The table lists only the elements of the chemical compounds for the thermo-chemical treatment, the oxide layer and the sputtering material.

The EDS analysis performed in Area 2 revealed an aluminium content of 43.4–51.6% and oxygen 46.8–51.9% for all the studied layers. The chemical composition of the aluminium oxide layers should include 52.92% Al and 47.08% O. Nevertheless, only EDS analysis conducted in the middle zone of the layer will show a chemical composition similar to the stoichiometric calculations. The chemical composition analyses were carried out in the near-surface zone of the layer (about 5 µm from the surface). Reducing the aluminium content and increasing the oxygen content along the thickness of the Al_2_O_3_ layer causes a change in the stoichiometry of the formed layer. Small amounts of carbon from 1.5–2.2% were also seen in all the layers. It is probably carbon with a C–H bond, associated with the absorption of organic acids from the electrolyte used in the anodizing process, which is consistent with study [27]. The presence of sodium is the result of the dissolution of this element during the electrolysis process (which is a component of the EN AW-5251 alloy, AlMg2) and passing to the electrolyte, and then building it into the nanostructure of the layers. The EDS analysis carried out in Area 1 showed gold in all the layers up to 30.4%, coming from the sputtering preparation used for the cross-sections. More carbon was also revealed in Area 1 than Area 2. This is due to the fact that in the subsurface zone, apart from carbon with a C–H bond, there is also carbon with a C=O bond, which is the result of its absorption from the atmosphere, and is also consistent with work [27]. Area 1, in the case of the thermo-chemically treated layers, also included the resulting sub-layer. The analysis performed for these layers indicated the presence of Na, S, and Cr. They are elements included in the Na_2_SO_4_·10H_2_O and Na_2_Cr_2_O_7_·2H_2_O solutions used in the modification of the Al_2_O_3_ layers, which may also indicate the build-up of the compounds from these solutions.

### 3.4. Influence of Thermo-Chemical Treatment on Micromechanical Properties

Microhardness is one of the most important features of anodic oxide layers related to their mechanical properties and wear resistance. The microhardness of Al_2_O_3_ layers obtained by hard anodizing is the highest at the substrate and decreases at the layer surface owing to the dissolving effect of the electrolyte. The results of the *H_IT_* microhardness tests carried out on the surface of the layers (Table 4) revealed an increase in microhardness after thermo-chemical treatment in distilled water and in the Na_2_SO_4_·10H_2_O solution. The thermo-chemical treatment performed in the Na_2_Cr_2_O_7_·2H_2_O solution reduced the microhardness of the layers. The table also includes the values of the maximum indentation depth *h*, which facilitate interpretation of the changes in microhardness as a result of thermo-chemical treatment.

The increase in the microhardness of the layer after thermo-chemical treatment in water results from swelling of the aluminium oxide fibers. This leads to closure of the pores and transformation of the oxide into hydrated forms of Al_2_O_3_, most probably γ-AlOOH boehmite, formed at a temperature above 353 K. The formation of a boehmite sub-layer leads to significant development of the surface associated with an increase in roughness (Figure 2b and Figure 6b). The increase in the microhardness of the layer as a result of using the Na_2_SO_4_·10H_2_O solution in the thermo-chemical process may be caused, on the one hand, by the significant sorption of the of Na_2_SO_4_·10H_2_O solution layer and, on the other hand, by the formation of the thinnest (Table 2) but compact sub-layer (Figure 1c). The ratio of the sub-layer thickness to the maximum indenter indentation depth is 0.43 for the layer modified with distilled water, and 0.26 for the layer modified with the Na_2_SO_4_·10H_2_O solution. It follows that in the case of both samples, the microhardness tests concerned de facto the sub-layer-layer system, with a much larger share of the Al_2_O_3_ layer. The reduction in the microhardness of the layer as a result of using the Na_2_Cr_2_O_7_·2H_2_O solution in the thermo-chemical process can be related to the fact that the ratio of the sub-layer thickness to the maximum penetration depth is 0.99. This means that in this case the tests only concern the microhardness of the sub-layer. 

### 3.5. Adhesive Properties of Layers after Thermo-Chemical Treatment

**Figure 3:**
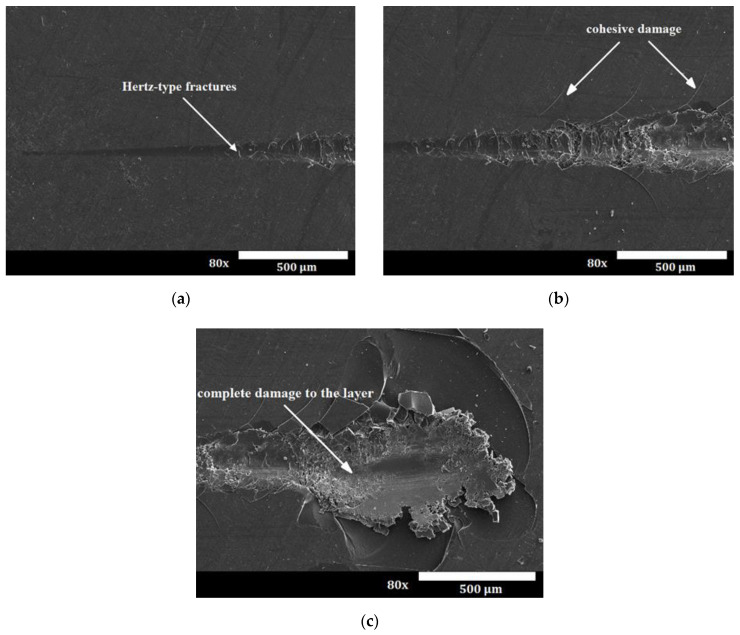
Damage to tested layers: (**a**) Hertz cracks, (**b**) cohesive damage, (**c**) adhesive damage and complete damage to layers.

An optimal composite surface layer intended for sliding contacts should be characterized by good adhesion to the substrate, durability and good tribological properties. The most popular method of testing the adhesion strength of the layer with the substrate is a scratch test. As a result of the test, a scratch on the surface of the layer is obtained, which may have the character of elastic Hertz cracks inside the scratch mark (Figure 3a), cohesive cracks (Figure 3b) and adhesive cracks (Figure 3c). Cohesive cracks result from the action of forces inside the torn layer and appear as cracks in the direction perpendicular to the direction of the indenter movement. Adhesive cracks result from the action of significant compressive stresses arising in the layer and are revealed as a result of crushing and tearing off the layer in front of the indenter.

**Figure 4:**
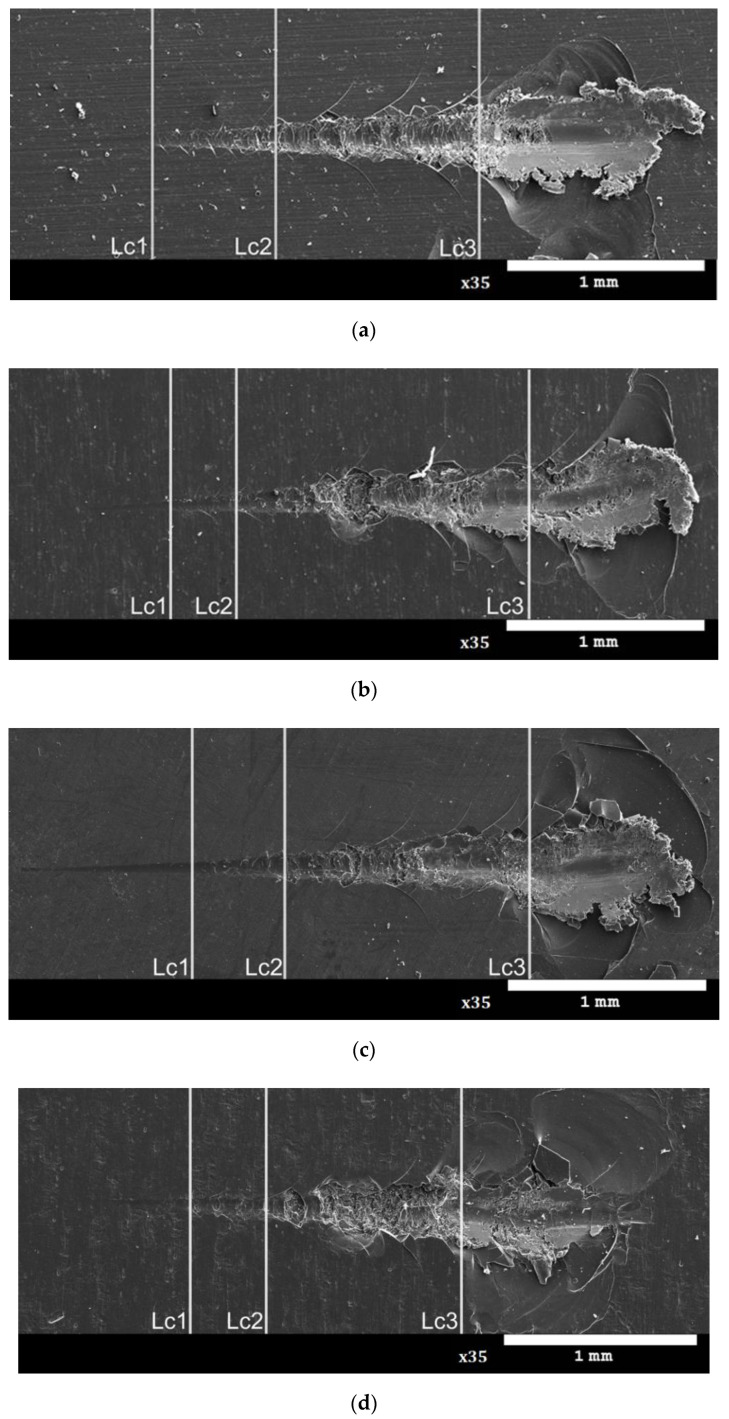
Critical loads determined for layers: (**a**) Sample 1, (**b**) Sample 2, (**c**) Sample 3, (**d**) Sample 4; *Lc1*-Hertz cracks, *Lc2*-cohesive cracks and *Lc3*-adhesive cracks and complete damage of layer.

As a result of the tests, scratches with all three types of cracks were obtained. The places where the Hertz cracks, cohesive and adhesive cracks occurred, along with complete damage to the layer, are shown in Figure 4. The values of critical loads *Lc1*, *Lc2* and *Lc3* are presented in Table 5. The analysis of the test results showed that the reference layer is characterized by the lowest scratch resistance (no sub-layer). The first damage was found at load *Lc1*, the value of which was 3.7 N. For this layer, the earliest among those tested, adhesive cracks and complete abrasion of the layer were also demonstrated, revealing the base material under the *Lc3* load of 19.93 N.

**Figure 5:**
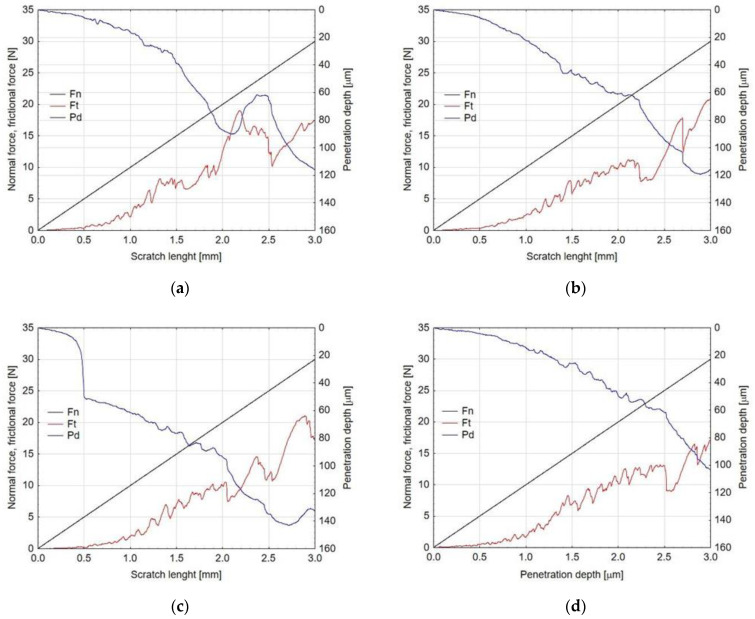
Characteristics of scratch test parameters (**a**) Sample 1, (**b**) Sample 2, (**c**) Sample 3, (**d**) Sample 4; *F_n_*–normal force, *F_t_*–frictional force, *P_d_*–penetration depth.

The Al_2_O_3_ layer is characterized by the best scratch resistance after thermo-chemical treatment in the Na_2_SO_4_·10H_2_O solution, for which the highest values were attained, practically for all the critical loads. The smallest thickness of the sub-layer and the highest value of its microhardness translate into higher mechanical resistance *Lc3* of 22.51 N, which also indicates good adhesion of the sub-layer-layer system. In the case of the thermo-chemical process carried out in distilled water and the Na_2_Cr_2_O_7_·2H_2_O solution, complete damage to the layers occurred by penetration of the indenter under the loads of *Lc3* of 22.75 N and 21.28 N, respectively, which also suggests improvement in the mechanical resistance. The parameters recorded during the scratch tests of the layers allowed the characteristics of the friction force and the penetration depth of the indenter under the load to be plotted (Figure 5). 

As the load increases, the friction force rises and the penetration depth of the indenter increases. In the graphs, it is only possible to analyse the influence of the load on the scratch parameters of the sub-layer-layer system. The thickness of the sub-layer itself was only 0.37–1.77 µm, depending on the thermo-chemical treatment compound used, which was only 0.01% of the penetration depth scale in the graph. A sharp increase in friction force Ft was recorded on all the graphs at the indenter penetration depth of approximately 60 µm. This phenomenon resulted from direct cooperation of the indenter with the surface of the aluminium alloy because the thickness of the Al_2_O_3_ layers produced in the anodizing process was 48–50 µm. The lowest values of the friction forces and the penetration depth of the indenter were exhibited by the layer after thermo-chemical treatment in the Na_2_Cr_2_O_7_·2H_2_O solution. This was probably the result of the good sliding properties of the sub-layer as a result of the applied thermo-chemical treatment. 

### 3.6. Geometric Structure of Surface of Layers

**Figure 6:**
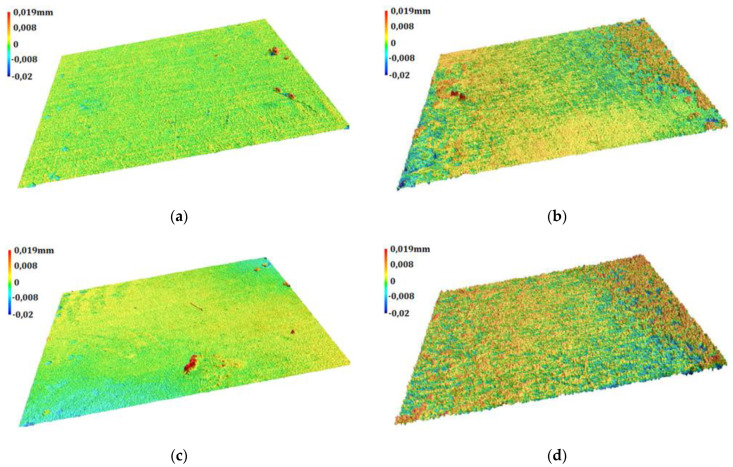
Axonometric projection of surface of examined layers: (**a**) Sample 1, (**b**) Sample 2, (**c**) Sample 3, (**d**) Sample 4.

The geometric structure of the surface was studied in order to determine the roughness of the Al_2_O_3_ layer and the sub-layers produced as a consequence of thermo-chemical treatment of the layers. The surface roughness can significantly affect the course of tribological phenomena of the cooperating elements of the kinematic pair; therefore, such tests are particularly important in the case of layers with a tribological application. The axonometric projection of the surface of the layers in the 3D approach (Figure 6) and the roughness parameters (Table 6) showed a significant influence of the thermo-chemical treatment process on the geometric structure of the surface. 

Axonometric projections reveal differences in the surface topography of the examined layers, which can be divided into two groups showing some similarities. The surface of the reference layer and the layer subjected to the thermo-chemical process in the Na_2_Cr_2_O_7_·2H_2_O solution display lower roughness and a higher surface load capacity than the layers modified in distilled water and the Na_2_Cr_2_O_7_·2H_2_O solution.

The test results reveal a favourable geometric structure of the surface of the oxide layer and the sub-layer produced in the process of thermo-chemical treatment in the Na_2_SO_4_·10H_2_O solution. This is indicated by the low values of the square deviation of the height of surface roughness from reference plane *Rq*. The negative values of asymmetry coefficient *Rsk* indicate the bearing character of these surfaces, and the low values of parameters *Rp*, *Rpk* and *Rsk* prove the low susceptibility of the surface to abrasion. The Al_2_O_3_ layers after thermo-chemical treatment with distilled water and the Na_2_Cr_2_O_7_·2H_2_O solution reveal an increase in surface roughness *Rq*. At the same time, a decrease in the density of the surface peaks is also observed, resulting from the increasing value of the average distance between the peaks of the *Rsm* profile. The sub-layer produced in the Na_2_Cr_2_O_7_·2H_2_O solution, apart from the highest roughness, is characterized by a lower load-bearing capacity, which is indicated by a positive *Rsk* value. The four-fold higher value of *Rk* than the reference sample indicates the lower abrasion resistance of this sub-layer. At the same time, the highest value of the *Rvk* parameter among the studied surfaces indicates a good ability to transfer the sliding material to the surface of the sub-layer, demonstrating self-lubricating properties in the case of cooperation with polymers.

## 4. Conclusions

On the basis of the research conducted and analysis of the results, it can be concluded that the Al_2_O_3_ layers subjected to the thermo-chemical treatment processes in distilled water, Na_2_SO_4_·10H_2_O and Na_2_Cr_2_O_7_·2H_2_O solutions are characterized by complete surface coverage of the applied treatment products. The precipitates are of a surface-structural character in the form of a new phase (sub-layer). The analysis of the chemical composition of the layers revealed the presence of elements included in the solutions used in the thermo-chemical treatment process, which proves that the compounds from these solutions are incorporated into the nanostructure of the layers. The thickness of the sub-layer depends on the compound used for the thermo-chemical treatment and was only 0.37 µm in the case of modification of the layer in the Na_2_SO_4_·10H_2_O solution, for which the highest microhardness (over 7 GPa) was recorded. This layer also exhibited the best scratch resistance and the highest surface load capacity among all the investigated layers. Despite the different properties of the sub-layer, the layer modified in the Na_2_Cr_2_O_7_·2H_2_O solution also seemed to be noteworthy. The low microhardness and high roughness testified to the low abrasion resistance of this sub-layer, which eliminated it from tribological cooperation, especially in heavily loaded friction pairs: however, it had the ability to transfer polymer sliding material, demonstrating self-lubricating properties.

## Figures and Tables

**Table 1:** Parameters of thermo-chemical treatment.

Sample	Thermo-Chemical Treatment		
	Compound for Thermo-Chemical Treatment	Solution Temperature *T* [K]	Process Time *t* [min]
1	-	371	60
2	H_2_O distillate		
3	Na_2_SO_4_·10H_2_O		
4	Na_2_Cr_2_O_7_·2H_2_O		

**Table 2:** Thickness of sub-layer obtained by thermo-chemical treatment.

Compound for Thermo-Chemical Treatment	Thickness of the Sub-Layer [μm]	Standard Deviation [μm]
H_2_O distillate	0.63	0.049
Na_2_SO_4_·10H_2_O	0.37	0.057
Na_2_Cr_2_O_7_·2H_2_O	1.77	0.088

**Table 3:** Chemical composition shown in cross-sections of oxide layers.

Sample	Chemical Element % by Mass						
	Al	O	Au	C	Na	S	Cr
				**Area 1**			
1	49.9	43.5	5.3	1.3	-	-	-
2	25.7	30.5	30.4	8.4	-	0.5	1.3
3	44.0	49.7	2.2	3.0	-	1.0	-
4	26.5	46.4	11.9	4.3	6.4	-	1.0
				**Area 2**			
1	51.6	46.8	-	1.7	-	-	-
2	45.9	51.9	-	2.2	-	-	-
3	43.4	51.7	-	2.0	1.8	1.1	-
4	47.3	47.1	-	1.5	3.8	-	-

**Table 4:** Microhardness of studied layers and maximum indentation depth.

Compound for Thermo-Chemical Treatment	*H_IT_* [GPa]	Standard Deviation [GPa]	*h* [μm]	Standard Deviation [μm]
layer without treatment	4.475	0.634	1.669	0.109
H_2_O distillate	6.278	1.498	1.443	0.150
Na_2_SO_4_·10H_2_O	7.109	2.981	1.390	0.264
Na_2_Cr_2_O_7_·2H_2_O	3.848	0.674	1.789	0.139

**Table 5:** Critical loads demonstrated for studied layers.

Compound for Thermo-Chemical Treatment	*Lc1* [N]	*Lc2* [N]	*Lc3* [N]
layer without treatment	3.70	9.44	19.93
H_2_O distillate	4.42	6.45	22.75
Na_2_SO_4_·10H_2_O	6.24	11.07	22.51
Na_2_Cr_2_O_7_·2H_2_O	5.91	9.78	21.28

**Table 6:** Roughness parameters of studied layers.

Compound for Thermo-Chemical Treatment	*Rq* [μm]	*Rsk* [−]	*Rsm* [μm]	*Rk* [μm]	*Rpk* [μm]	*Rvk* [μm]
layer without treatment	1.304	−0.071	88.742	3.104	1.447	1.497
H_2_O distillate	3.059	−0.240	100.811	7.213	3.063	3.803
Na_2_SO_4_·10H_2_O	1.011	−0.244	86.734	2.261	1.287	1.266
Na_2_Cr_2_O_7_·2H_2_O	4.744	0.019	120.111	12.918	4.059	3.891

## Data Availability

The data presented in this study are available on request from the corresponding author. The data are not publicly available due to insufficient space to insert data.
